# Suppression of autophagy through JAK2/STAT3 contributes to the therapeutic action of rhynchophylline on asthma

**DOI:** 10.1186/s12906-020-03187-w

**Published:** 2021-01-07

**Authors:** Hui Li, Qianyu Bi, Hongxia Cui, Chuanfeng Lv, Meng Wang

**Affiliations:** 1 Department of Medical Affairs, Jining No. 1 People’s Hospital, Jiankang Road, Jining, Shandong 272011 People’s Republic of China; 2 Institute of Traditional Chinese Medicine, Shandong University of Traditional Chinese Medicine, Jinan, Shandong 250355 People’s Republic of China; 3 Department of Oncology, Jining No. 1 People’s Hospital, Jining, Shandong 272011 People’s Republic of China; 4 Department of Pharmacology, Jining No. 1 People’s Hospital, Jining, Shandong 272011 People’s Republic of China

**Keywords:** Autophagy, Asthma, Rhynchophylline, JAK2, STAT3

## Abstract

**Background:**

Asthma is a chronic inflammatory disease characterized by airway remodeling and inflammation. Rhynchophylline is a kind of indole alkaloid isolated from *Uncaria rhynchophylla*. Here we investigated the effect of rhynchophylline on autophagy in asthma.

**Methods:**

A mice model of asthma was established by ovalbumin challenge. Histopathological changes were assessed by hematoxylin-eosin staining, periodic acid-schiff staining and Masson staining. The levels of IgE in serum, interleukin-6 and interleukin-13 in bronchoalveolar lavage fluid, as well as the activities of superoxide dismutase and catalase in lung tissues were detected. The expression of autophagy-related genes and Janus kinase (JAK) 2/ signal transducer and activator of transcription (STAT) 3 signal was detected by western blot and immunofluorescence. Airway smooth muscle cells (ASMCs) were isolated, and the effect rhynchophylline on autophagy in ASMCs was explored.

**Results:**

Our data showed that rhynchophylline treatment alleviated inflammation, airway remodeling, and oxidative stress in asthma. In addition, autophagy, which was implicated in asthma, was suppressed by rhynchophylline with decreased level of autophagy-related proteins. Furthermore, rhynchophylline suppressed the JAK2/STAT3 signaling pathway, which was activated in asthma. In vitro study showed that rhynchophylline suppressed ASMC autophagy through suppressing the activation of JAK2/STAT3 signal.

**Conclusions:**

Our study demonstrated that rhynchophylline can alleviate asthma through suppressing autophagy in asthma, and that JAK2/STAT3 signal was involved in this effect of rhynchophylline. This study indicates that rhynchophylline may become a promising drug for the treatment of asthma.

## Background

Asthma is a chronic inflammatory disease characterized by cough, breathlessness and wheeze. This complex disease is usually triggered by genetic or environmental factors, such as allergen exposure and infections. An estimated 300 million people suffer from asthma worldwide [1]. Over the past decades, the incidence and prevalence of asthma is increasing, especially in children. There is an urgent need for novel therapeutic strategies.

Autophagy, an evolutionarily conserved process, is initiated with the formation of autophagosome sequestering misfolded proteins or damaged organelles, and then fused with lysosome for subsequent degradation. It also plays an important role in the survival of cells suffering from starvation, due to autophagy acts as a process for self-digesting to supply nutrients for the synthesis of substance needed for survival [2]. Multiple autophagy-related genes, such as microtubule associated protein 1 light chain 3 (LC3), beclin-1 and autophagy related genes (ATGs), are implicated in asthma, and polymorphism of these genes is associated with asthma [3]. Due to its role in asthma, by regulating autophagy, ketamine or simvastatin mitigates asthma [4, 5].

*Uncaria rhynchophylla*, a traditional Chinese medicine, is usually used in traditional Chinese medicine formulas for the treatment of asthma. Rhynchophylline (Rhy), a kind of indole alkaloid isolated from *Uncaria rhynchophylla*, shows anti-inflammation and anti-oxidant capabilities [6, 7]. Our previous study showed that rhynchophylline can mitigate inflammation in asthma, attenuate hypertrophy of bronchial smooth muscle cells [8], however, the effect of rhynchophylline on autophagy in asthma remains unknown.

Signal transducer and activator of transcription (STAT) 3, one of the transcription factor STAT family, is involved in multiple biological processes. Growing evidence reveals that STAT3 plays a crucial role in asthma. Activation of STAT3 is found in peripheral blood mononuclear cells and airway smooth muscle tissues from asthma patients [9] and correlated with the increased cytokines [10]. Inhibition of STAT3 signaling can remit the airway inflammation [11], inhibit the proliferation of airway smooth muscle cells (ASMCs) and angiogenesis [9], and regulate IgE produce [12]. However, whether STAT3 signaling pathway could mediate the function of rhynchophylline on asthma remains unclear.

In this study, we investigated the effects of rhynchophylline on autophagy and STAT3 signal in asthma. Our study revealed that STAT3 signal mediates the function of rhynchophylline on autophagy in asthma.

## Methods

### Materials

Ovalbumin (OVA) was obtained from Sigma (St.Louis, MO, USA). Rhynchophylline was obtained from Melonepharma (Dalian, China) or Aladdin (Shanghai, China). Transforming growth factor (TGF)-β1 was obtained from USCN (Wuhan, China). WP1066 was obtained from Aladdin. Mouse interleukin (IL)-6 enzyme-linked immunosorbent assay (ELISA) kit and IL-13 ELISA kit were obtained from BOSTER (Wuhan, China) and IgE ELISA kit was obtained from Multi Science (Hangzhou, China). Catalase (CAT) detection kit and superoxide dismutase (SOD) detection kit were obtained from JianchengBio (Nanjing,China). Primary antibodies against LC3, ATG5, p62, Janus kinase (JAK) 2, p-JAK2 and STAT3 were obtained from Abclonal (Wuhan, China). Beclin-1 antibody and β-actin antibody were obtained from Santa Cruz (Dallas, TX, USA). P-STAT3 antibody was obtained from Affinity (Changzhou, China). Histone H3 antibody was obtained from ABGENT (San Diego, CA, USA). α-SMA antibody was obtained from Abcam (Cambridge, UK). Calponin antibody was obtained from Proteintech (Wuhan, China). Horse radish peroxidase-labeled, Cy3-labelled and fluorescein isothiocyanate (FITC)-labeled secondary antibodies were obtained from Beyotime (Shanghai, China).

### Animal experimental protocol

Female BALB/c mice (8-week-old, weighting 22–24 g) were obtained from Liaoning Changsheng Biotechnology co., Ltd. (China, Benxi), fed in a standard condition (12 h-light/12 h-dark cycles, temperature 22 ± 1 °C, humidity 45–55%), and accessed to food and water freely. The mice were randomized into four groups: control, OVA, OVA+Rhy 40, OVA+Rhy 80. Mice in the OVA, OVA+Rhy 40, OVA+Rhy 80 groups were sensitized with 20 μg OVA (companied with 1 mg aluminum hydroxid; intraperitoneal injection) on Day 0, 14, 28 and 42. Then the mice were challenged with 1% OVA for 30 min, three times a week, from Day 21 to Day 42. Mice in the control group received equal amount of phosphate buffered saline (PBS). Mice in the OVA+Rhy 40 group and OVA+Rhy 80 group received 40 mg/kg or 80 mg/kg Rhy (intragastric administration) at 30 min before OVA challenge. Mice in the control and OVA group received equal amount of vehicle. 24 h after the last OVA challenge, mice in each group were euthanized by injection with pentobarbital sodium (200 mg/kg, intraperitoneal injection). The lung tissues, serum and bronchoalveolar lavage fluid were collected. All animal experiments were conduct following the Care and Use of Laboratory Animal and approved by the Ethics Committee of Jining No. 1 People’s Hospital.

### Isolation of ASMCs

ASMCs were isolated from the bronchia tissues of a 5-week-old mouse. The bronchia tissues were obtained in sterile environment and ASMCs were isolated as described previously [8]. The isolated ASMCs were grown in DMEM (Gibco, Grand Island, NY, USA) containing 10% fetal bovine serum (Biological Industries, Cromwell, CT, USA) and cultured in a cell incubator with 5% CO_2_. Immunofluorescence with calponin and α-SMA antibodies was performed to identify the isolated ASMCs. The isolated ASMCs were calponin-positive and α-SMA-positive. ASMCs were treated with or without TGF-β1 (5 ng/ml), Rhy (10 μM or 20 μM) or WP1066 (3 μM) for 24 h. Then the cells were harvested for subsequent experiments.

### Histological examination

The lung tissues of mice in each group were fixed, dehydrated, embedded in paraffin and cut into 5 μm slices. The slices were deparaffinated, rehydrated, and then subjected to routine hematoxylin-eosin (HE) staining, Masson’s trichrome staining, or periodic acid-schiff (PAS) staining. Images were captured under a microscope (BX53, OLYMPUS, Tokyo, Japan). Inflammation was assessed by an 8-point semiquantitative scoring system [13, 14]. The number of PAS-positive cells was recorded.

### Wright-Giemsa staining

Bronchoalveolar lavage fluid from each group was collected and stained with Wright-Giemsa staining solution (JianchengBio). The number of inflammatory cells, neutrophils, monocytes, eosinophils and lymphocytes was recorded.

### Elisa

IgE in the serum of mice in each group was detected with a mouse IgE ELISA kit according to the instruction. The levels of IL-6 and IL-13 in the bronchoalveolar lavage fluid and the level of IL-6 in the medium of ASMCs after different treatment were detected with corresponding ELISA kits.

### Western blot

Total proteins in each group were extracted using lysis buffer with 1 mM phenylmethanesulfonyl fluoride (Beyotime) on ice and centrifuged at 10000×g at 4 °C for 5 min. Nuclear proteins and plasma proteins were extracted with a nucleoprotein extraction kit (Beyotime). The concentration of proteins was measured with a BCA protein assay kit (Beyotime). Equal amount of proteins from each group were separated by sodium dodecyl sulfate polyacrylamide gel electrophoresis and then transferred onto polyvinylidene fluoride membranes (Millipore, Bedford, MA, USA). After blockade, the membranes were incubated with primary antibodies: LC3 (1: 1000), beclin-1 (1: 200), ATG5 (1: 1000), p62 (1: 1000), JAK2 (1: 1000), p-JAK2 (1: 1000), STAT3 (1: 500), p-STAT3 (1: 1000), β-actin (1: 1000) and Histone H3 (1: 2000) overnight at 4 °C. After washing with Tris buffered saline with Tween, the membranes were incubated with corresponding secondary antibodies (1: 5000). The blots were visualized with an enhanced chemiluminescence substrate luminescence kit (Beyotime). Images were captured and analyzed with a Gel-Pro-Analyzer software.

### Activities of SOD and CAT

The activities of CAT and SOD in the lung tissues or cells were detected with corresponding kits according to the instruction.

### Immunofluorescence

After deparaffination and rehydration, the slices of lung tissues from each group were washed in PBS and restored in antigen retrieval solution. Then the slices were blocked with goat serum (Solarbio, Beijing, China) and then incubated with primary antibodies: beclin-1 (1: 50) or STAT3 (1: 100) at 4 °C overnight. Then the slices were incubated with Cy3-labelled or FITC-labeled secondary antibodies (1: 200) at room temperature for 60 min, following by washing with PBS. Thereafter, the slices were stained with 4′,6-diamidino-2-phenylindole (DAPI, Aladdin). Images were captured under a fluorescence microscope (OLUMPUS).

For immunofluorescence of cells, the cells were grown on coverslips and fixed with 4% paraformaldehyde. Following by washing with PBS, the cells were permeabilized with 0.1% TritonX-100, followed by blocking with goat serum. Thereafter, the cells were incubated with primary antibodies: Calponin (1: 100), α-SMA (1: 100), STAT3 (1: 100), followed by incubating with Cy3-labelled secondary antibodies (1: 200). Then the cells were stained with DAPI.

### Dansylcadaverine (MDC) staining

Cells were grown on coverslips. After indicated treatment, the cells were stained with MDC (KeyGen, Nanjing, China) at room temperature for 15 min. After washing, the cells were observed under a fluorescence microscope.

### CCK-8 assay

The isolated ASMCs were seeded into a 96-well plate in quintuplicate. After indicated treatment for 24 h, 10 μl CCK-8 (Sigma) was added into each well. After incubation for 1 h, the absorbance at 450 nm was measured with a microplate reader (BIOTEK, Winooski, VT, USA).

### Statistical analysis

GraphPad Prism 8.0 was used for statistical analysis. Results of this study were presented as mean ± standard deviation (SD). Differences between groups were analyzed using Kruskal-Wallis test followed by Dunn’s multiple comparisons as the post hoc or One-way Analysis of Variance followed by Tukey’s multiple comparisons as the post hoc. *P* < 0.05 was considered as significant.

## Results

### Rhy mitigates inflammation in asthmatic mice

**Fig. 1:**
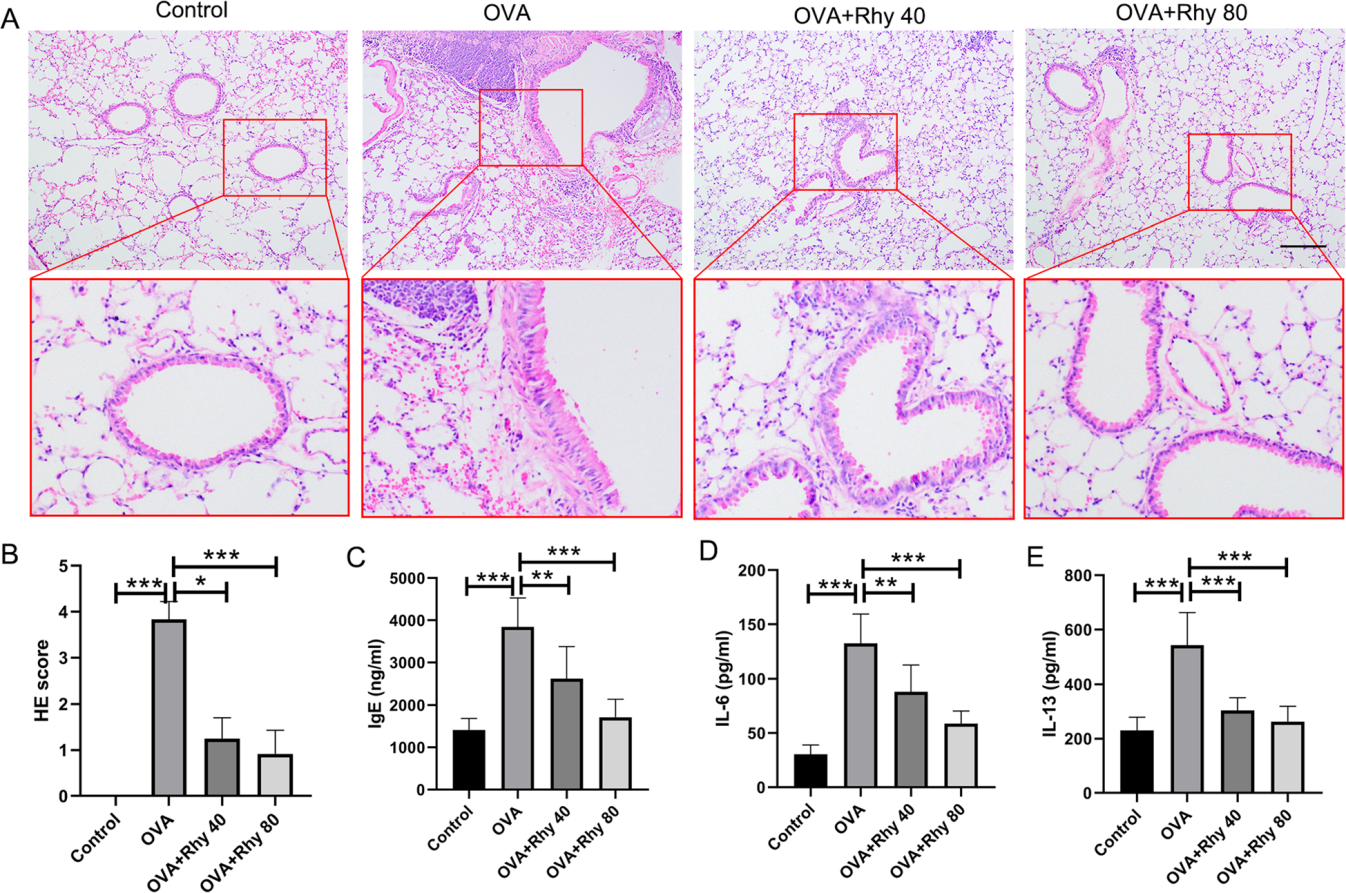
Rhy mitigates inflammation in asthmatic mice. Asthma models were established by OVA challenge and treated with 40 or 80 mg/kg Rhy. (**a**) The histopathological changes in lung tissues from each group were assessed by HE staining. Bar = 200 μm. (**b**) Inflammation scores in each group. (**c-e**) The levels of IgE in serum, IL-6 and IL-13 in bronchoalveolar lavage fluid were detected by ELISA. *N* = 6 for each group. The results were presented as mean ± SD. * *p* < 0.05, ** *p* < 0.01, *** *p* < 0.001. OVA, ovalbumin; Rhy, rhynchophylline; HE, hematoxylin-eosin; IL, interleukin; ELISA, enzyme-linked immunosorbent assay; SD, standard deviation

Inflammation is one of the hallmarks of asthma. After treatment with 40 or 80 mg/kg Rhy, the histopathological changes of lung tissues were assessed by HE staining. Rhy alone showed no toxicity to the lungs (supplementary Fig. 1a-c). In OVA group, there was obvious inflammatory cells infiltration in bronchus. However, upon treatment with Rhy, these changes were mitigated (Fig. 1a). Consistently, the inflammation scores of lung tissues from the OVA group were higher than that from the control group. While the inflammation scores of lung tissues from mice treated with Rhy were decreased compared with the OVA group (Fig. 1b). Additionally, the number of inflammatory cells in bronchoalveolar lavage fluid was assessed. The number of total inflammatory cells as well as neutrophils, monocytes, eosnophils and lymphocyte was all increased in the OVA group. However, after treatment with Rhy, all these numbers were declined compared with the OVA group (Table 1). Furthermore, the elevated IgE, which is a hallmark of asthma, in the OVA group was reduced upon Rhy treatment (Fig. 1c), indicating a mitigation effect of Rhy on asthma. The levels of IL-6 and IL-13 were also detected by ELISA. The levels of these two inflammatory cytokines were increased in the OVA group compared with the control group, and were later decreased upon Rhy treatment (Fig. 1d-e). These results suggested that Rhy has a mitigatory effect on asthma.

**Table 1:** Inflammatory cells in the bronchoalveolar lavage fluid

	Control	OVA	OVA+Rhy 40	OVA+Rhy 80
Inflammatory cells (×10^5^)	1.63±0.38	4.71±0.95 ^***^	2.54±0.53	2.08±0.47 ^#^
Neutrophil (×10^4^)	1.16±0.32	7.13±1.59 ^***^	3.24±0.72	2.61±0.58
Monocytes (×10^4^)	9.68±2.88	19.90±5.89 ^*^	12.85±2.94	11.10±3.31
Eosnophils (×10^4^)	1.00±0.21	8.49±0.22 ^***^	3.06±0.77	2.33±0.52 ^#^
Lymphocyte (×10^4^)	4.05±1.09	10.66±2.16 ^**^	5.59±1.28	4.48±1.09 ^##^

N=6. * *p*< 0.05, ** *p*< 0.01, ***p < 0.001 compared with Control; # *p*< 0.05, ## *p*< 0.01 compared with OVA.

### Rhy mitigates airway remodeling and oxidative stress in asthmatic mice

**Fig. 2:**
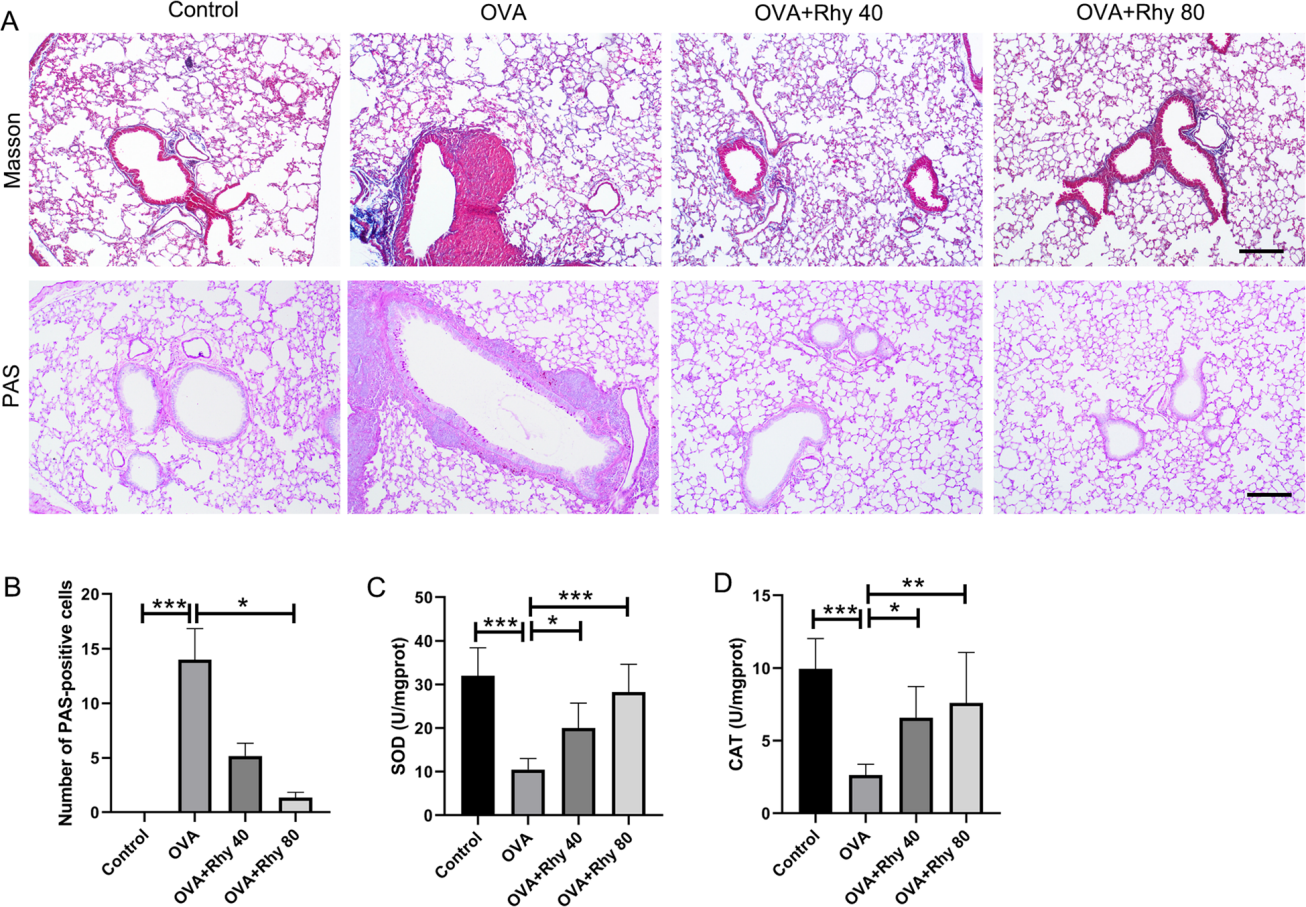
Rhy mitigates airway remodeling and oxidative stress in asthmatic mice. **a** After treatment with Rhy, the lung tissues were subjected to Masson’s trichrome staining and PAS staining. Bar = 200 μm. **b** Number of PAS-positive cells. **c-d** Activities of SOD and CAT. N = 6 for each group. The results were presented as mean ± SD. * *p* < 0.05, ** *p* < 0.01, *** *p* < 0.001. Rhy, rhynchophylline; SOD, superoxide dismutase; CAT, catalase; SD, standard deviation

Airway remodeling, which is characterized by goblet cell hyperplasia and collagen deposition, is another hallmark of asthma. To elevate the effect of Rhy on airway remodeling, the lung tissues from each group were stained with Masson’s trichrome staining and PAS staining. The results showed that in the OVA group, there was large amount extracellular matrix deposition in the lung tissues. However, after treatment with Rhy, the extracellular matrix deposition induced by OVA challenge was reduced (Fig. 2a). Goblet cells in each group were stained with PAS. There were more PAS-positive, mucous-containing goblet cells in the OVA group, which was reduced by Rhy treatment (Fig. 2a). Consistent with the results of PAS staining, the upheaval of PAS-positive cell number in the OVA group was decreased upon Rhy treatment (Fig. 2b). In addition, the level of oxidative stress in asthma was also assessed by the activities of SOD and CAT. In the OVA group, the activities of SOD and CAT were lower than those in the control group. Whereas, upon treatment with Rhy, the activities of SOD and CAT were increased significantly compared with the OVA group (Fig. 2c-d), indicating that Rhy also ameliorated the oxidative stress status of asthma.

### Rhy inhibits autophagy in asthmatic mice

**Fig. 3:**
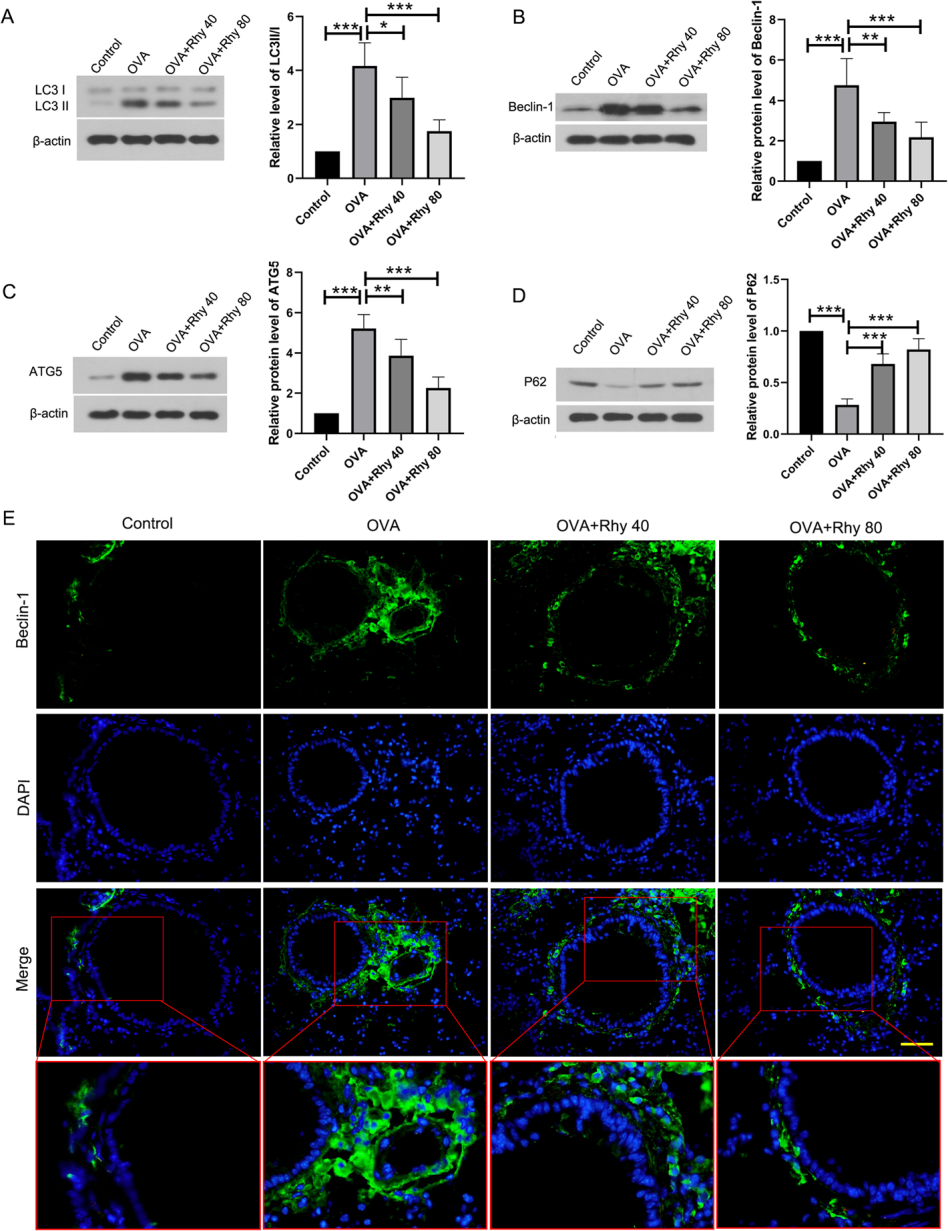
Rhy inhibits autophagy in asthmatic mice. **a-d** After treatment with Rhy, the levels of LC3, beclin-1, ATG5, and p62 were detected by western blot. **e** Level of beclin-1 in the bronchus was elevated by immunofluorescence. Bar = 50 μm. Green fluorescence, beclin-1; blue fluorescence, DAPI. N = 6 for each group. The results were presented as mean ± SD. * *p* < 0.05, ** *p* < 0.01, *** *p* < 0.001. Rhy, rhynchophylline; LC3, microtubule associated protein 1 light chain 3; ATG5, autophagy related 5; SD, standard deviation

To evaluate the status of autophagy in asthmatic mice, the levels of autophagy-related genes, such as LC3, beclin-1, ATG5 and P62, in each group were detected by western blot. In the OVA group, there was an obvious increase in the levels of LC3 II compared with the control group (Fig. 3a). Also, the levels of beclin-1 and ATG5 were increased (Fig. 3b-c) and the level of P62 was decreased (Fig. 3d) in the OVA group, indicating that autophagy was activated in asthma. However, after treatment with Rhy, the levels of LC3 II, beclin-1 and ATG5 were decreased and the level of P62 was increased. The level beclin-1 in lung tissues was also elevated by immunofluorescence. There was much more beclin-1 (green fluorescence) in the OVA group, compared with the control group. After treatment with Rhy, the level of beclin-1 was decreased and showed less green fluorescence comparing with the OVA group (Fig. 3e). These results suggested that autophagy activated in asthma was inhibited by Rhy treatment.

### Rhy inhibits the JAK2/STAT3 signaling pathway

**Fig. 4:**
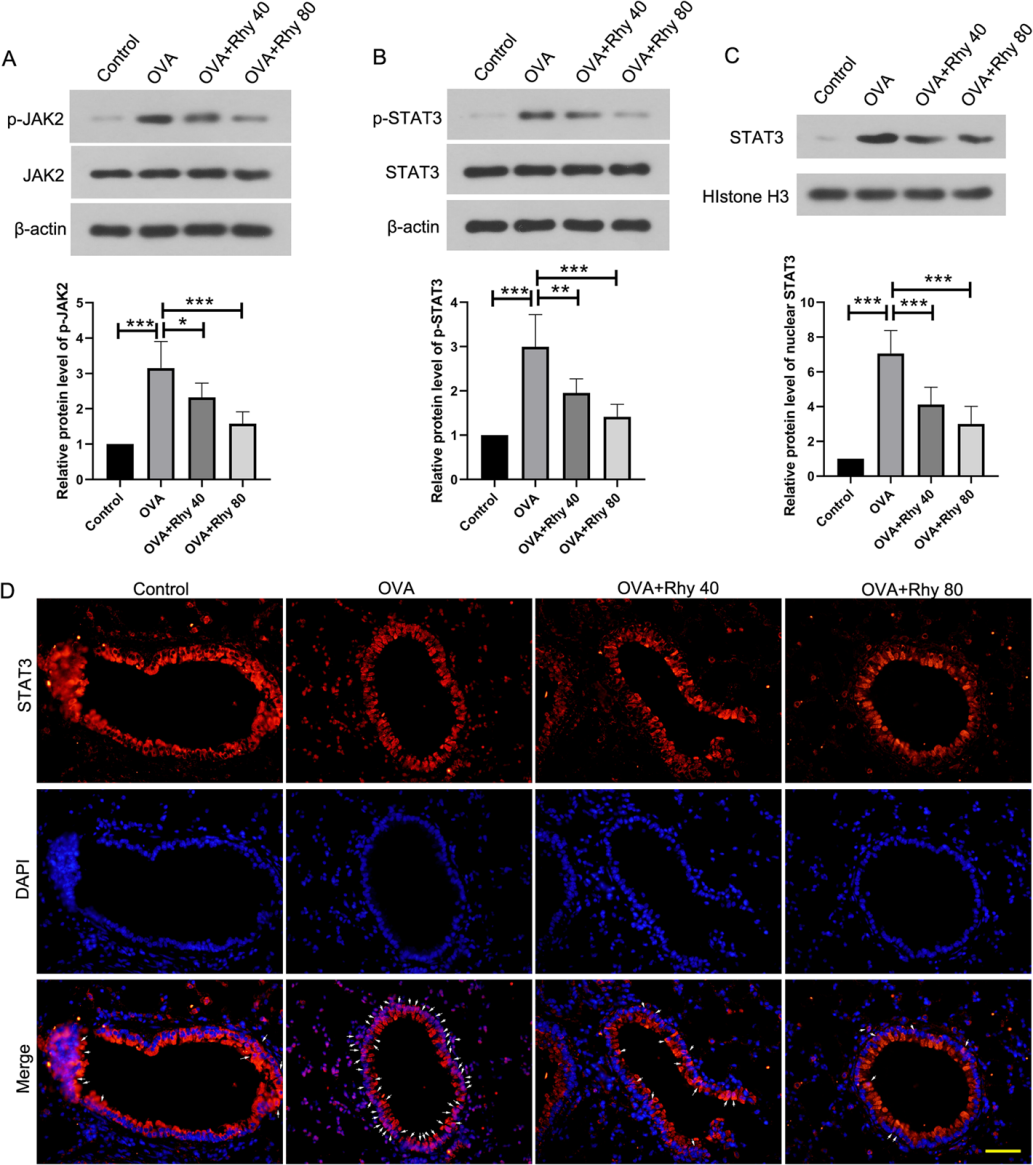
Rhy inhibits the JAK2/STAT3 signaling pathway. **a-c** After treatment with Rhy, the levels of JAK2, p-JAK2, STAT3, p-STAT3 and nuclear STAT3 were detected by western blot. **d** Level of STAT3 in the bronchus was elevated by immunofluorescence. Bar = 50 μm; White arrows indicate nuclear STAT3. Red fluorescence, STAT3; blue fluorescence, DAPI. N = 6 for each group. The results were presented as mean ± SD. * *p* < 0.05, ** *p* < 0.01, *** *p* < 0.001. Rhy, rhynchophylline; JAK2, janus kinase 2; STAT3, signal transducer and activator of transcription 3; SD, standard deviation

To investigate whether the JAK2/STAT3 signaling pathway was involved in the effect of Rhy, the activation of JAK2/STAT3 signaling pathway in each group was detected by western blot. As showed in Fig. 4, in the OVA group, the phosphorylated JAK2 and phosphorylated STAT3 were increased, compared with the control group, indicating the activation of JAK2/STAT3 signaling pathway in asthma. However, after treatment with Rhy, the levels of phosphorylated JAK2 and phosphorylated STAT3 were decreased significantly (Fig. 4a-b). Moreover, the level of nuclear STAT3 was increased in the OVA group, but was reduced by Rhy treatment (Fig. 4c). In addition, the nuclear translocation of STAT3 was elevated by immunofluorescence. In the OVA group, there was more STAT3 in the nuclear, which was reduced by Rhy treatment (Fig. 4d). These results suggested that Rhy inhibited the activation of JAK2/STAT3 signaling pathway in asthmatic mice.

**Fig. 5:**
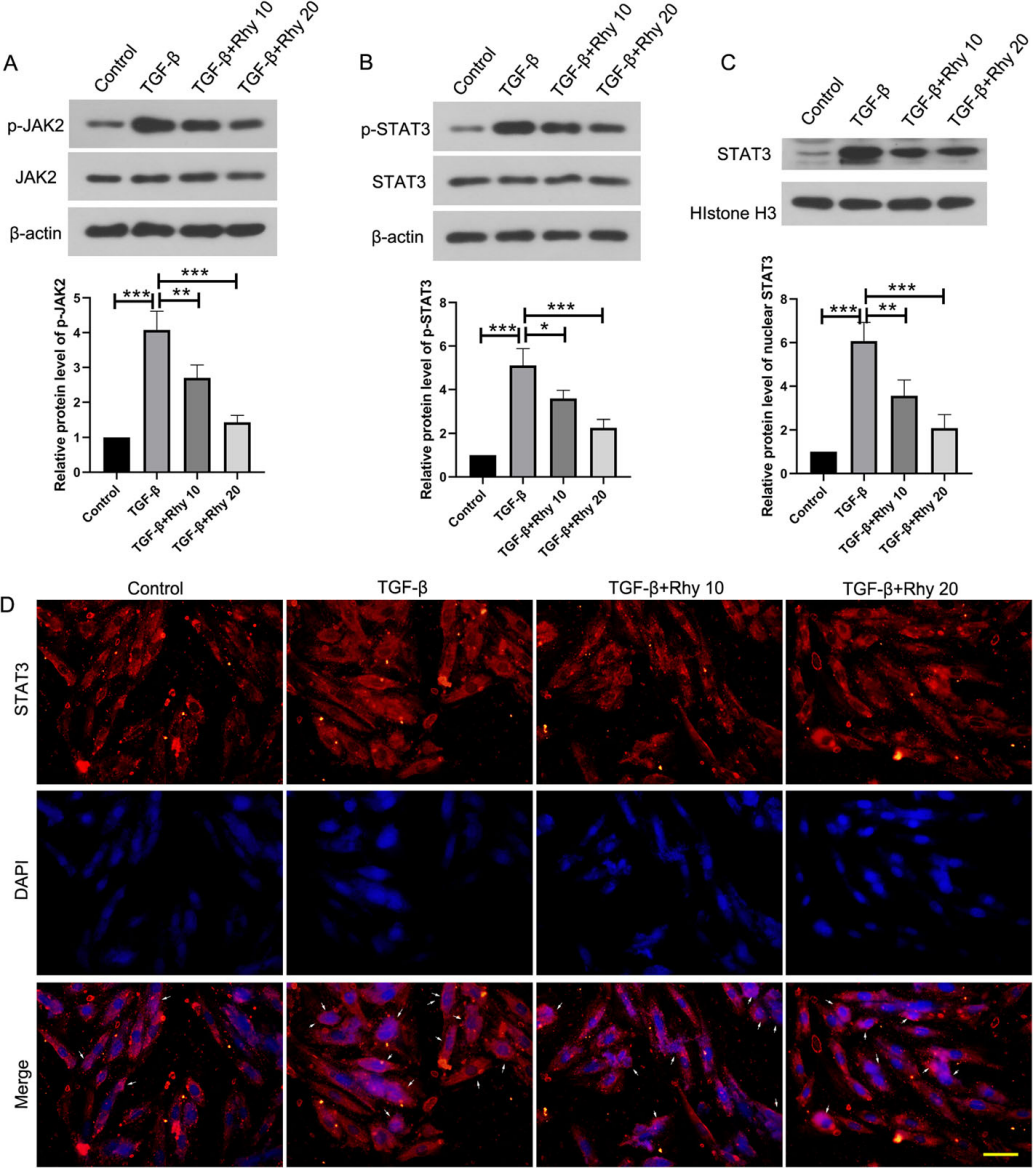
Rhy inhibits the activation of JAK2/STAT3 signaling pathway in TGF-β treated ASMCs. **a-c** After treatment with TGF-β and Rhy, the levels of JAK2, p-JAK2, STAT3, p-STAT3 and nuclear STAT3 were detected by western blot. **d** The level of STAT3 in ASMCs was elevated by immunofluorescence. Bar = 50 μm; White arrows indicate nuclear STAT3. Red fluorescence, STAT3; blue fluorescence, DAPI. The results were presented as mean ± SD. * *p* < 0.05, ** *p* < 0.01, *** *p* < 0.001. Rhy, rhynchophylline; JAK2, janus kinase 2; STAT3, signal transducer and activator of transcription 3; ASMCs, airway smooth muscle cell; SD, standard deviation

ASMCs were isolated and identified by immunofluorescence with calponin and α-SMA antibodies (Supplementary Fig. 2). Thereafter, ASMCs treated with TGF-β were used as an in vitro model of asthma. Treatment with Rhy alone showed no effect on the viability of ASMCs (Supplementary Fig. 1d). The phosphorylated JAK2 and phosphorylated STAT3 in the cells treated with TGF-β were increased compared with the control cells, indicating the activation of JAK2/STAT3 signaling in the in vitro model of asthma. However, after treatment with Rhy, the phosphorylated JAK2 and phosphorylated STAT3 was decreased (Fig. 5a-b), indicating the inhibitory effect of Rhy on the JAK2/STAT3 signaling pathway. The level of STAT3 in nuclear, which indicates the activation of this signaling pathway, was increased significantly in TGF-β-treated cells, but reduced upon Rhy treatment (Fig. 5c). The translocation of STAT3 was also evaluated by immunofluorescence. Consistently, cells treated with TGF-β showed more STAT3 in the nuclear compared with the control cells. Whereas, Rhy treatment decreased the nuclear STAT3 level (Fig. 5d). These data demonstrated that Rhy inhibited the JAK2/STAT3 signaling pathway in cells treated with TGF-β.

### Rhy suppresses autophagy through JAK2/STAT3 signaling pathway

**Fig. 6:**
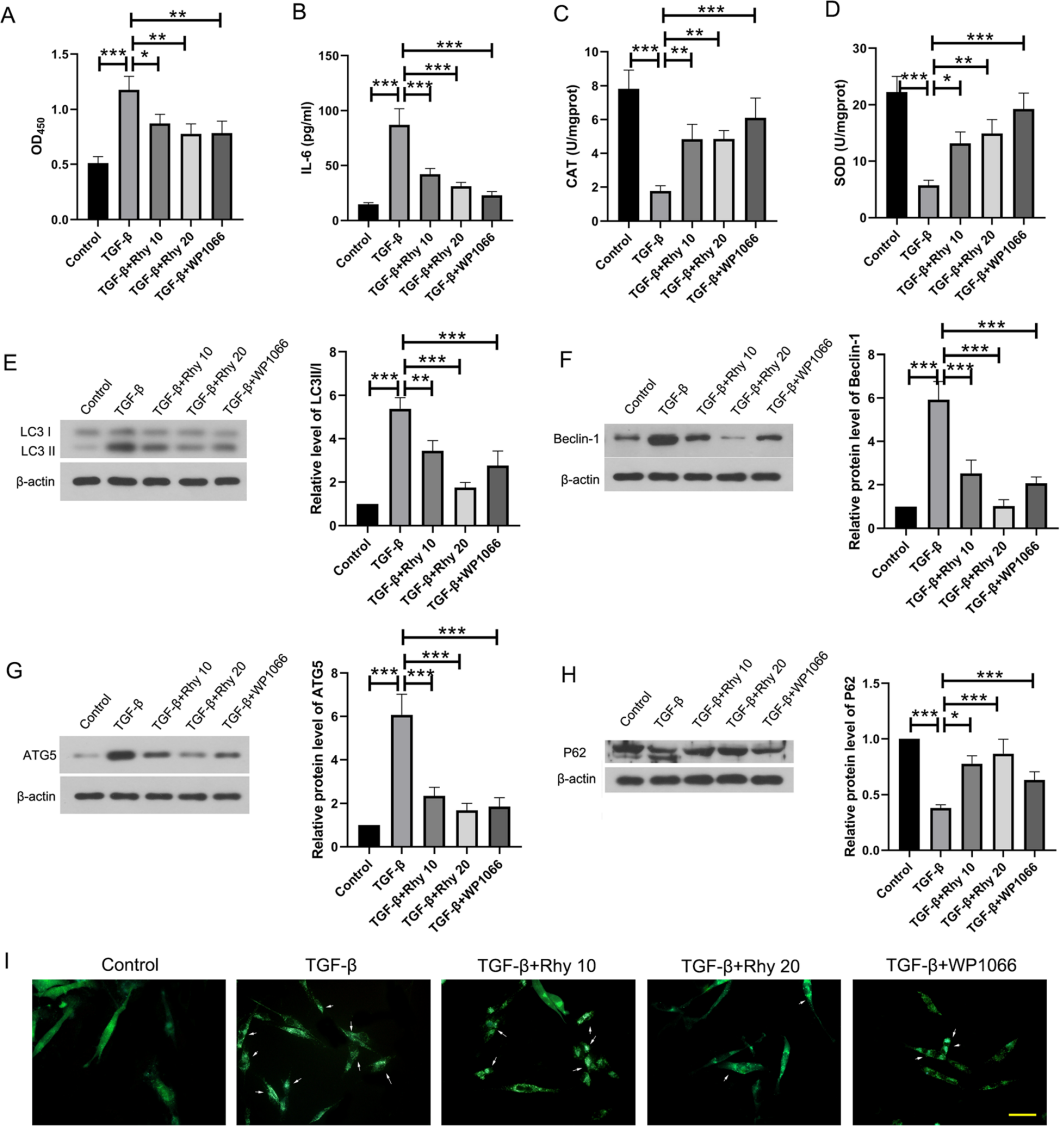
Rhy mitigates asthma through inhibiting JAK2/STAT3 signaling pathway. **a** After treatment with TGF-β and Rhy or WP-1066, CCK-8 assay was performed to assess the proliferation of ASMCs. **b-d** The level of IL-6 and activities of SOD and CAT were detected. **e-h** The levels of LC3, beclin-1, ATG5 and p62 were detected by western blot. (**i**) MDC staining was performed to assess autophagy in ASMCs. Bar = 100 μm. The results were presented as mean ± SD. * *p* < 0.05, ** *p* < 0.01, *** *p* < 0.001. Rhy, rhynchophylline; JAK2, janus kinase 2; STAT3, signal transducer and activator of transcription 3; ASMC, airway smooth muscle cell; IL, interleukin; SOD, superoxide dismutase; CAT, catalase; LC3, microtubule associated protein 1 light chain 3; ATG5, autophagy related 5; SD, standard deviation

**Fig. 7:**
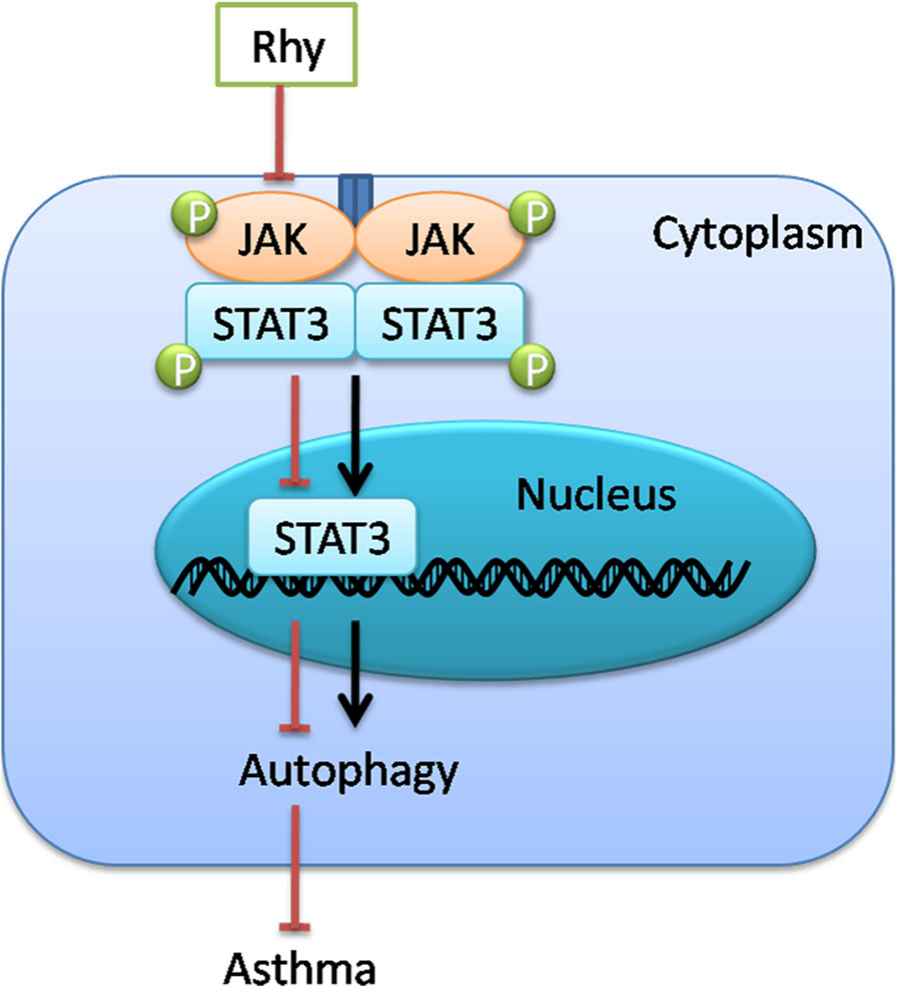
Schematic diagram for the function of rhynchophylline

To confirm whether JAK2/STAT3 signaling pathway mediates the effect of Rhy on autophagy, WP1066, an inhibitor of the STAT3 signaling pathway, was employed in this study. Thereafter, the cell activity was assessed by CCK-8. Proliferation of ASMCs induced by TGF-β was reduced by Rhy treatment, which was similar to that of WP-1066 treatment (Fig. 6a). Additionally, in ASMCs, the increased level of IL-6 induced by TGF-β, and the activities of SOD and CAT decreased by TGF-β, were reversed by Rhy treatment (Fig. 6b-d). These results demonstrated that Rhy inhibited TGF-β induced proliferation and IL-6 level, but boost the activities of SOD and CAT, and these effects were similar to those of WP-1066. Furthermore, autophagy markers in ASMCs were also detected. The levels of LC3 II, beclin-1, and ATG5 was increased, and the level of P62 was decreased in cells treated with TGF-β. However, after treatment with Rhy, these changes were reversed, which were similar to those of WP-1066 (Fig. 6e-h). Additionally, MDC staining showed that autophagy induced by TGF-β was reduced by Rhy treatment (Fig. 6i). These results suggested that Rhy suppressed autophagy in asthma, maybe through inhibiting the JAK2/STAT3 signaling pathway (Fig. 7).

## Discussion

In the present study, we explored the effect of rhynchophylline on asthma both in vivo and in vitro. Rhynchophylline alleviated inflammation and ameliorated airway remodeling in asthma through suppressing autophagy. Further study indicated that the JAK2/STAT3 signaling pathway may mediate the function of rhynchophylline on autophagy.

Results in this study and our previous study [8] showed that rhynchophylline had an therapeutic effect on asthma. Rhynchophylline reduced inflammation and ameliorated airway remodeling in asthma. Similarly, rhynchophylline also shows an anti-inflammation effect in tourette syndrome and lipopolysaccharide-induced inflammation [6, 15]. In addition, the disordered antioxidant system, including SOD and CAT, in asthma was enhanced upon rhynchophylline treatment, indicating that rhynchophylline may also improve the disordered oxidative stress in asthma. Consistently, rhynchophylline can also decrease the markers of oxidative stress, including myeloperoxidase, malondialdehyde and reactive oxygen species, and enhanced the Nrf2 signaling pathway, a famous antioxidative signaling pathway [16]. Also, its analog Y396 can protect endothelium from oxidative stress-induced dysfunction [17].

Airway smooth muscle hyperplasia is implicated in airway remodeling in asthma. Our previous study showed that rhynchophylline inhibited the proliferation of ASMCs in asthma through the TGF-β/Smad and mitogen-activated protein kinase signals [8]. Herein, our study showed that suppressing autophagy of ASMCs also contributed to the effect of rhynchophylline on asthma. Autophagy is noted in bronchial tissues of asthma. It is indicated that autophagy is essential for the production of collagen and fibronectin in ASMCs [18]. Inhibition of autophagy in asthma can mitigate airway hypertension and inflammation [19]. In our study, we showed that autophagy, which was enhanced in OVA-challenged mice and TGF-β treated ASMCs, was suppressed by rhynchophylline. We hypothesized that suppression of autophagy by rhynchophylline may contribute to its therapeutic action on asthma. Interestingly, rhynchophylline can protect endothelial progenitor cells against senescence during hypertension [20], indicating that rhynchophylline may also has a protective effect on endotheliocytes. As endothelial dysfunction is also implicated in the pathogenesis of asthma, the protective effect of rhynchophylline on endotheliocytes may also contribute to its role in asthma. Additional researches may be carried out to reveal the pharmacological action of rhynchophylline on endotheliocytes.

In this study, we showed that the phosphorylation level of JAK2 and STAT3 was decreased after treatment with rhynchophylline both in vivo and in vitro, with less STAT3 in the nucleus, indicating that rhynchophylline suppressed the activation of JAK2/STAT3 signaling pathway. We hypothesized that JAK2/STAT3 signal may mediate the effect of rhynchophylline on asthma. STAT3 signal is usually activated in asthma and plays an important role in disease process. Suppression of STAT3 signal was reported to prevent lung inflammation and remodeling [11]. Recently, Hongyan et al. also showed the inhibition effect of rhynchophylline on the activation of JAK2/STAT3 signaling pathway in Tourette syndrome rats [21]. Interestingly, rhynchophylline alone had no effect on JAK2/STAT3 signaling pathway in control rats, indicating that rhynchophylline alone may have no toxicity. Actually, we confirmed that rhynchophylline showed no toxicity both on the lungs of control mice and ASMCs. This prompts us that rhynchophylline may have an amelioration effect on asthma patients, but with no toxicity in healthy population.

## Conclusion

In this study, we revealed that rhynchophylline suppressed autophagy of ASMCs through inhibiting the JAK2/STAT3 signaling pathway, which may contribute to its therapeutic action on asthma. However, there are still some limitations in our study. First, rhynchophylline shows an amelioration effect on asthma mice, whereas, its effect on asthma patients needs to be verified. Secondly, this study focus on smooth muscle cells, while other types of cells, such as endotheliocytes, fibroblasts and inflammatory cells, play critical roles in asthma. The effects of rhynchophylline on these cells remain unclear and further investigation is needed.

## Supplementary Information

**Additional file 1.** Supplementary Figures

## Data Availability

The datasets used and analysed during the current study are available from the corresponding author on reasonable request.

## Abbreviations

ASMC: Airway smooth muscle cell
ATG: Autophagy related gene
CAT: Catalase
DAPI: 4′,6-diamidino-2-phenylindole
ELISA: Enzyme-linked immunosorbent assay
FITC: Fluorescein isothiocyanate
HE: Hematoxylin-eosin
IL: Interleukin
JAK: Janus kinase
LC3: Microtubule associated protein 1 light chain 3
MDC: Dansylcadaverine
OVA: Ovalbumin
PAS: Periodic acid-schiff
PBS: Phosphate buffered saline
Rhy: Rhynchophylline
SD: Standard deviation
SOD: Superoxide dismutase
STAT: Signal transducer and activator of transcription
TGF: Transforming growth factor;
