# Supplementary material for: Suppression of autophagy through JAK2/STAT3 contributes to the therapeutic action of rhynchophylline on asthma
Source: BMC Complement Med Ther. 2021 Jan 7;21:21. doi: 10.1186/s12906-020-03187-w (PMC7792286; doi:10.1186/s12906-020-03187-w)
Supplement: Supplementary file 1 — Additional file 1. Supplementary Figures [file 12906_2020_3187_MOESM1_ESM.pdf]

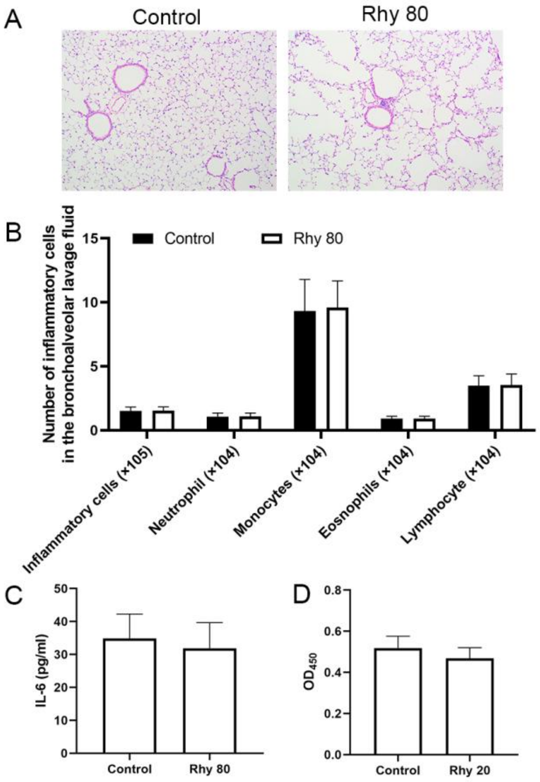

Supplementary Figure 1. Rhy showed no toxicity to lung or airway smooth muscle cells. After treatment with/without rhynchophylline, the histopathological changes in lung tissues were assessed by HE staining (A), the number of inflammatory cells in broncholveolar lavage fluid (B), and the level of IL-6 in broncholveolar lavage fluid (C) were recorded. (D) The cell viability of airway smooth muscle cells were assessed by CCK-8 assay after treatment with rhynchophylline.

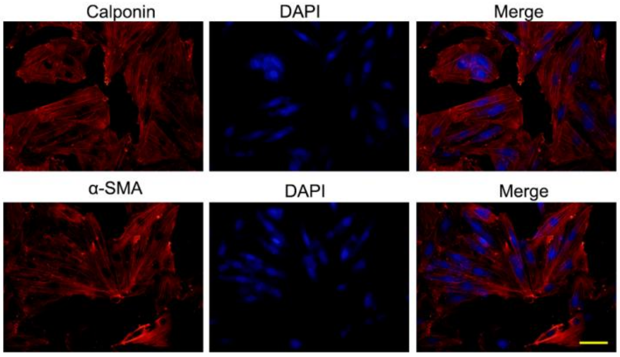

Supplementary Figure 2. Identification of the isolated ASMCs. The isolated ASMCs were identified

by immunofluorescence with calponin antibody and α-SMA antibody. Bar= 50 μm. Red fluorescence, calponin or α-SMA; blue fluorescence, DAPI. The isolated ASMCs were calponin-positive and α-SMA-positive
